# Wine: An Aspiring Agent in Promoting Longevity and Preventing Chronic Diseases

**DOI:** 10.3390/diseases6030073

**Published:** 2018-08-08

**Authors:** Eleni Pavlidou, Maria Mantzorou, Aristeidis Fasoulas, Christina Tryfonos, Dimitris Petridis, Constantinos Giaginis

**Affiliations:** 1 Department of Food Science and Nutrition, University of the Aegean, Myrina, 81400 Lemnos, Greece; athanarist@aegean.gr (A.F.); ch.trifon@aegean.gr (C.T.); cgiaginis@aegean.gr (C.G.); 2 Department of Food Technology, Technological Educational Institute of Thessaloniki, 57400 Sindos, Greece; petridis@food.teithe.gr

**Keywords:** wine, vine, diet, health, dementia, cardiovascular disease

## Abstract

Introduction: Moderate wine consumption is a characteristic of the Mediterranean diet. Studies around the world have shown a beneficial effect of moderate alcohol intake, especially wine, on health. This review aims to critically summarise the most recent studies that investigate the beneficial effects of moderate wine intake on human health. Methods: The PubMed database was comprehensively searched to identify trials published from 2013 to 2018 that investigated the association between moderate wine consumption and health. Results: The most recent studies confirm the valuable role of moderate wine consumption, especially red wine, in the prevention and treatment of chronic diseases such as cardiovascular disease, metabolic syndrome, cognitive decline, depression, and cancer. In the meantime, recent studies also highlight the beneficial role of red wine against oxidative stress and in favour of desirable gut bacteria. The beneficial role of red wine has been attributed to its phytochemical compounds, as highlighted by clinical trials, where the effect of red wine has been compared to white wine, non-alcoholic wine, other alcoholic drinks, and water. Conclusions: Moderate wine intake, at 1–2 glasses per day as part of the Mediterranean diet, has been positively associated with human health promotion, disease prevention, and disease prognosis.

## 1. Introduction

Moderate wine consumption is a characteristic of the Mediterranean diet, and was first described by Ancel Κeys in the Seven Countries study. High adherence to this dietary model is associated with reduced risk for various chronic diseases [1,2,3,4,5]. Moderate alcohol consumption, especially wine [6], is generally regarded to be beneficial to health [7,8]. Other alcoholic drinks, such as beer, also seem to improve the lipid profile and other factors related to atherosclerosis [9].

In fact, the “French paradox” [10] suggests that red wine consumption is the reason for lower ischaemic heart disease mortality in the French population, where high saturated fat consumption is observed [10], although some scientists support that the benefit should be attributed to alcoholic drinks in general [11].

Despite certain health benefits, alcohol consumption has been considered detrimental for public health in general [12]. Additionally, binge drinking [7] and high alcohol intake [13] have also been associated with negative health impacts. In fact, 88,000 deaths in the United States each year are attributed to excessive alcohol intake, representing 1 in 10 deaths among adults aged 20–64 years old [14]. Although moderate alcohol consumption reduces cardiovascular mortality, individual characteristics and dietary habits should be taken into account, as decreases in alcohol consumption are related to general health benefits [13].

Alcohol consumption guidelines vary between countries [11]. In the USA, alcohol consumption is recommended not to exceed one drink per day for women and two drinks per day for men, where one drink is described to contain 14 g of pure alcohol [15]. In the United Kingdom, it is recommended that both men and women drink no more than 14 units alcohol per week, where one unit is equivalent to 10 mL of pure alcohol [16]. Guidelines highlight that people who do not drink alcohol should not be advised to start drinking, while alcohol consumption should be part of a healthy eating pattern [17].

Over the last decades, disease patterns have changed, with non-communicable diseases being on the increase and the communicable diseases on the decrease. Current diseases, such as cardiovascular diseases, cognitive impairment, and cancer, and their prevention have been associated with environmental factors such as diet and lifestyle. Alcohol and wine have been studied over the years with respect to the health benefits and risks their consumption confers.

Several epidemiological and clinical studies have attributed wine (specifically its phytochemical components such as resveratrol, quercetin, polyphenols, and flavonoids) as having positive effects on health, health promotion, disease prevention, and disease prognosis [8,11].

However, the 15-year experience gained from the Seven Countries Study, which enrolled 11,579 healthy men aged 40–59 years old and recorded 2,289 deaths due to coronary heart disease (CHD), suggests the significance of the investigation of many other factors for safe conclusions. Factors such as age, smoking, serum cholesterol, blood pressure, body mass index (BMI), and physical activity play a very important role. There are also some other important factors, such as socioeconomic status or ethnic differences, which are often not taken into account and can affect diet and other aspects of life [18]. Thus, findings should be taken cautiously.

The present study aims to critically collect and summarise in depth the most recent clinical data to highlight the association between wine consumption and health.

## 2. Methods

Observational studies and clinical trials investigating the relation between wine consumption and health were thoroughly searched in the PubMed database using relative key words for studies published from 2013 to April 2018. The key words were, “wine health benefits”, “wine” and “cardiovascular disease”, “blood pressure”, “metabolic syndrome”, “weight”, “cancer”, and “mental health”. Only studies conducted in humans were taken into account. The primary search resulted in more than 120 publications, which were then evaluated. The secondary search was restricted to clinical trials, reviews, and meta-analyses written in English with titles including wines and selected diseases. Finally, 54 studies fulfilling the criteria were selected. Studies that were focused on the influence of alcohol on the human body and those focused only on the biochemical ingredients of grapes were excluded. Thus, 65 other studies, referring to the impact of moderate wine consumption, were taken into account and summarized.

## 3. Results

### 3.1. Association between Wine Consumption and Cardiovascular Disease 

In the last five years, 10 studies have investigated the role of wine consumption on cardiovascular disease (Table 1).

Levantesi et al. [19] aimed to investigate the associations of wine intake, cardiovascular events and total mortality in 1,248 patients after myocardial infarction, in subjects enrolled in the Gruppo Italiano per lo Studio della Sopravvivenza nell’Infarto Miocardico (GISSI)-Prevenzione Trial. Moderate wine consumption at ≤500 mL/day was associated with lower risk of cardiovascular events and mortality, as compared to not drinking wine after 3.5 years of follow-up [19].

In 6973 patients with chronic heart failure enrolled in the GISSI-Heart Failure (GISSI-HF) trial, moderate wine consumption of one glass per day was associated with a better health status according to the Kansas City Cardiomyopathy Questionnaire, lower depressive symptoms according to the Geriatric Depression Scale, and lower vascular inflammation, yet without improved 4-year clinical outcomes [20]. However, in 449 older (75.7 ± 8.2 years old) U.S. male physicians with prevalent heart failure who were followed for 7 years, a J-shaped association between alcohol consumption and mortality was observed, with the lowest mortality at 1–2 drinks per day independent of alcoholic drink type (beer, wine, or liquor) [21].

In patients with type 2 diabetes, the association between alcohol consumption and cardiovascular disease is not clear. Blomster et al. [22] undertook a study to investigate the role of moderate alcohol consumption on cardiovascular health. After 5 years of follow-up, compared to those who did not drink alcohol, those who had moderate alcohol consumption had fewer cardiovascular events and microvascular complications, and lower all-cause mortality. Those who mainly drunk wine benefited the most [22].

Apostolidou et al. [23] studied the effects of moderate wine consumption in 40 otherwise healthy individuals with high cholesterol. In this crossover study participants consumed either “tannat” red wine or a placebo drink (125 mL for women and 250 mL for men daily) for one month. The antioxidant capacity and vitamin E levels of both patients with high cholesterol and subjects with normal cholesterol were improved, while in patients with high cholesterol the fasting LDL/HDL ratio was also improved [23]. Chiu et al. [24] investigated the effect of red wine extract of onion and red wine on cardiovascular disease risk factors. Twenty-three hypercholesteraemic participants were randomised to consume 250 mL daily of red wine or red wine extract of onion for 10 weeks. As a result, the body’s antioxidant capacity was increased, delaying the oxidation of LDL cholesterol. However, compared to red wine, the red wine onion extract had an additional hypocholesterolaemic effect, lowering total and LDL cholesterol while modulating inflammation and factor VII, and showing a better cardioprotective effect [24]. 

The role of wine consumption on platelet aggregation against platelet-activating factor was investigated in a small cross-over study with 12 healthy men [25]. Participants consumed a standardised meal along with either white wine, red wine, ethanol, or water. A significant effect was found in platelet sensitivity against the platelet-activating factor, with greater effect after red wine consumption, compared to both ethanol and water. The plasminogen activator inhibitor-1 concentration was higher for all alcoholic beverages compared to water, while triglycerides were only increased significantly after ethanol consumption as compared to water. Red and white wine lowered postprandial triglyceride concentrations [25]. In the Vino Veritas randomised trial the effects of red and white wine on atherosclerosis were investigated [26]. In this study, 157 healthy participants were randomised to receive either white or red wine for one year. After 12 months, LDL was similarly decreased in both groups, while total cholesterol was lowered in the red wine group, but the levels did not differ from the white wine group. In general this study failed to show positive clinical differences in atherosclerosis markers in healthy participants [26].

In patients with carotid atherosclerosis, the combination of a polyphenol-rich Mediterranean diet and moderate daily physical activity (30 min/day) with moderate red wine consumption (100 mL for women and 200 mL for men) for 20 weeks did not affect the middle cerebral and internal carotid blood flow velocity [27]. The absence of significant improvement may be due to the fact that 66% of the study population was on statin therapy [27]. However, in the same patient population, these lifestyle changes (healthy diet plus red wine, and exercise) for 20 weeks did improve the LDL/HDL ratio of the participants [28].

The review by Fernández-Solà [13] refers to epidemiological studies, case–control studies, and meta-analyses that indicate a U-type dose-dependent bimodal relationship between alcohol consumption (mainly wine and beer) and cardiovascular health. On the basis of these analyses, the reductions of cardiovascular events and mortality are associated only with low to moderate alcohol consumption, as compared to abstinence. This relationship was also reported in the 4th century BC, by Hippocrates, to emphasize the harmful effects of alcohol abuse on the heart. It is further argued that initiation of alcohol consumption for the benefit of health should not be encouraged, as a substantial number of diseases are associated with the harmful effects of high alcohol consumption. The beneficial and negative effects of alcohol consumption, as well as the overall lifestyle of the individual (smoking, lack of exercise, eating habits, etc.) should always be taken into account and evaluated [13].

### 3.2. Association between Wine Consumption and Blood Pressure

Seven interventional studies have been undertaken since 2013 to study the effect of wine consumption and blood pressure (Table 2).

In healthy women the dose-dependent association between wine consumption and blood pressure was investigated by Mori et al. [29]. Healthy premenopausal women were randomised to receive red wine in higher or lower levels than usual, or dealcoholized red wine for 4 weeks. When consuming higher volumes of wine than usual both diastolic and systolic blood pressure were elevated. This study showed that, like in men, 200–300 mL red wine per day was found to increase the 24-h diastolic and systolic blood pressure, as compared to dealcoholized wine [29].

Mori et al. [30] undertook another small randomised three-period crossover study in 24 patients with well-controlled type 2 diabetes [30], and examined the role of wine consumption on cardiovascular risk factors. Women were randomised to drink 230 mL/day red wine and men 300 mL/day red wine, or equivalent volumes of dealcoholized red wine or water, for 4 weeks. Red wine increased awake systolic and diastolic blood pressure, as compared to water. Diastolic blood pressure during sleep was decreased after red wine compared to dealcoholized red wine. Red wine increased the heart rate while subjects were sleeping, awake, and overall over 24 h, as compared to water and dealcoholized red wine. Compared to dealcoholized red wine, red wine did not affect glycaemic control or cardiovascular risk factors [30]. Gepner et al. [31] in their study had different results. In 54 participants (age = 57 years; 85% men) with type 2 diabetes who did not drink alcohol, a daily consumption of 150 mL red wine at dinner combined with a Mediterranean diet for 6 months did not change the median 24-h blood pressure, but did lower the blood pressure 3–4 h after wine consumption, at midnight (3–4 h after ingestion). In subjects homozygous for the gene encoding the ADH1B*2 variant that leads to fast alcohol metabolism, the median 24-h blood pressure and pulse pressure values were decreased compared to heterozygotes and those homozygous for the ADH1B*1 variant (slow alcohol metabolisers) [31]. In another 2-year-long study by Gepner et al. [32] in alcohol-abstaining patients with well-controlled type 2 diabetes, blood pressure remained unchanged after wine consumption initiation at 150 mL per day along with dinner [32].

In healthy subjects, two glasses of wine lead to a higher heart rate during the consumption, and lowered arterial compliance after consumption [33]. Eighteen healthy volunteers received one drink with alcohol (two glasses of red wine) and one drink without alcohol on two consecutive, but separate days. Red wine increased heart rate during alcohol ingestion, and reduced arterial compliance after ingestion. The day and ingestion period were found to have significant effects on heart rate, diastolic blood pressure and QKD, suggesting that the differences in response among the ingestion periods depended on whether alcohol had been consumed that day. For the first time their study indicates the effect of alcohol on 24-h arterial stiffness in a healthy group of volunteers [33].

Although chronic wine consumption and hypertension have been correlated, vasodilation after wine ingestion has also been observed. In a study by Barden et al. [34] consumption of 375 mL red wine (41 g alcohol) or 375 mL non-alcoholic wine or water along with light dinner on three different days was investigated in order to assess the effects of wine on blood pressure. Red wine consumption led to a decrease of blood pressure the first 4 h and then an increase after 20 h. The vasoconstrictor 20-hydroxyeicosatrienoic acid (20-HETE) was reduced 2 h after the ingestion of either drink, but was increased within 24-h after red wine consumption. The time point for the lowest levels of 20-HETE, at 2 h after red wine ingestion, is the time point when the highest alcohol levels in the blood are measured, indicating an important homeostatic response [34].

Draijer et al. [35] undertook a double-blind placebo controlled crossover study in order to assess the effect of two grape extracts (grape/red wine and grape alone) on blood pressure and vascular function in 60 untreated, mildly hypertensive participants for 4 weeks. Both extracts had high concentrations of anthocyanins and flavonols, but the control drink (grape alone) was relatively poor in catechins and procyanidins. The 24-h ambulatory systolic and diastolic blood pressures were significantly lower in the grape-wine extract intervention as compared to placebo, predominantly during the day. Plasma concentrations of the vasoconstrictor endothelin-1 decreased by 10%, but other measures of vascular function were not affected. The control drink had no effect on blood pressure and on vascular function, indicating an important role for catechins and procyanidins on blood pressure [35].

### 3.3. Association between Wine Consumption and Metabolic Syndrome

With respect to metabolic syndrome (MS), eight studies investigated the impact of wine on MS and its constituents (Table 3).

The prospective, population-based, cohort study Hispanic Community Health Study/Study of Latinos (HCHS/SOL), with data from 15,905 participants showed that low and moderate wine consumption was independently associated with lower risk of metabolic syndrome than alcohol abstinence [36]. The LifeLines cohort study also indicated a protective effect of wine consumption against metabolic syndrome, as well as low HDL cholesterol [37], while in the Brazilian Longitudinal Study of Adult Health (ELSA-Brasil), participants who drank mainly wine at 1–4 drinks per week were at lower risk of having metabolic syndrome [38]. On the other hand, another cohort study in younger subjects, with mean age at 35.4 years, failed to show an association between wine consumption and risk of metabolic syndrome [39].

The cross-sectional Primary Prevention of Cardiovascular Disease with a Mediterranean Diet (PREDIMED) study [40] with 5801 elderly participants at high cardiovascular risk highlighted the protective effect of red wine consumption. Compared with non-drinkers, those who drink one or more glasses red wine per day were found to have 44% lower risk of MS, 41% lower risk of high waist circumference, 58% lower HDL cholesterol, 72% lower high blood pressure, and 33% higher fasting plasma glucose after adjustment for confounders, especially in women, participants <70 years old, and those who smoked or used to smoke. Hence, moderate red wine intake can lower the risk of metabolic syndrome in elderly patients at a high cardiovascular risk. [40]. Considering type 2 diabetes, the E3N-EPIC cohort study [41] evaluated the role of wine consumption on the risk of developing diabetes in 66,485 women. In overweight women, wine consumption was negatively associated with risk of diabetes, at a consumption of two or more drinks per day, as compared to alcohol abstainers [41].

Sixty-seven men at high risk of cardiovascular disease were randomised in a crossover trial to receive red wine (30 g alcohol/day), the equivalent amount of dealcoholized red wine, and gin (30 g alcohol/day) for 4 weeks [42]. After four weeks of red wine and dealcoholized red wine consumption, although fasting glucose did not change, the mean adjusted plasma insulin and Homeostatic model assessment Insulin Resistance (HOMA-IR) were decreased, indicating a beneficial role of polyphenols on insulin sensitivity. Both red wine and gin increased HDL cholesterol, as well as apolipoprotein A-I and A-II concentrations. Lipoprotein(a) levels were decreased after the red wine consumption [42].

Well-controlled type 2 diabetes patients who did not drink alcohol were randomised to receive 150 mL of water, white or red wine with dinner for 2 years, along with Mediterranean diet. Red wine significantly increased HDL cholesterol and apolipoprotein(a)1, and decreased the total/HDL cholesterol ratio, while reducing the number of metabolic syndrome components. When ethanol metabolism genotypes were taken into account, it was observed that slow ethanol metabolisers benefited from both white and red wine in terms of glycaemic control as compared to fast ethanol metabolisers. Thus, initiation of moderate wine intake, especially red wine, can help improve the cardio-metabolic risk in well controlled diabetes patients, while with respect to glycaemic control, both ethanol and red wine’s non-alcoholic constituents can be beneficial [32]. However, this trial failed to show improvements in blood pressure, adiposity, liver function, drug therapy, and symptomatology [32].

### 3.4. Association between Wine Consumption and Weight

Five studies investigated the role of wine intake on weight gain (Table 4).

Wine consumption did not affect weight change in patients with type 2 diabetes after initiating daily wine consumption for 2 years [32,43]. A cohort study in men also failed to show an association between wine consumption or increased wine consumption and weight change [44]. However, middle aged participants of the of increased body weight and waist circumference for middle-aged adults of the Melbourne Collaborative Cohort Study were less likely to have greater waist circumference and body weight when drinking low to moderate amounts of alcohol, including wine [45]. Mean BMI was lower in people who drank alcohol and wine daily than those who consumed alcohol less frequently, while for a given alcohol intake, wine (and beer) intake was inversely associated with BMI and waist circumference [46].

### 3.5. Association between Wine Consumption and Cancer

In the last five years, 15 studies have been published concerning the relationship between wine and cancer (Table 5).

Studies have shown an association between the increased consumption of alcohol (>3 glasses for men and >2 glasses for women) and an increased risk of many types of cancer (oral cavity, pharynx, larynx, oesophagus, liver, large intestine, and female breast) [47,48]. However, even moderate alcohol consumption was shown to result in an increased incidence of cancer by Klatsky et al. [49]. However, the authors note that it is likely that the self-reported alcohol consumption was underestimated, making it difficult to draw firm conclusions [49].

The role of lifetime alcohol consumption on invasive epithelial ovarian cancer risk was evaluated in a case–control study by Cook et al. [50]. With respect to wine, its consumption was associated with a lower risk of cancer compared to not drinking alcohol, while the association was stronger for red wine-only drinkers as compared to white wine drinkers. However, most women did drink both types of wine [50].

Concerning urothelial cell bladder cancer [51] and endometrial cancer risk [52], the EPIC cohort study did not show a significant association between wine consumption and these types of cancer. However, in the analysis by Fagherazzi et al. [53] on the role of alcohol on risk of adulthood breast cancer in 66,481 women from the French E3N-EPIC study showed that in the postmenopausal period, wine consumption increased the risk of breast cancer by 33% for more than two glasses per day of wine, as compared with non-drinkers. Risk of ER+/PR+ breast cancer subtypes was also influenced by high wine intake [53].

A prospective study [54] on alcohol consumption and risk of basal cell carcinoma with data from 167,765 women in the Nurses’ Health Study (NHS) and NHS II and 43,697 men in the Health Professionals Follow-Up Study showed that increased wine consumption was associated with increased basal cell cancer risk after adjustment for sun exposure and other skin cancer risk factors [54], whereas no associations were observed between alcohol, red or white wine intake and risk of early-onset basal cell carcinoma in a case–control study [55]. Kubo et al. [56] examined the association between alcohol consumption and risk of melanoma and non-melanoma skin cancers in 59,575 white postmenopausal women enrolled in the Women’s Health Initiative Observational Study. After 10 years of follow up, and after adjusting for confounders such as sun exposure and skin type, it was observed that those who consumed more than seven drinks per week were in greater risk of both melanoma and non-melanoma skin cancers. Additionally, compared to non-drinkers, higher lifetime alcohol consumption of white wine or liquor was associated with an increased hazard of both cancers [56]. White wine was also found to increase the risk of melanoma by 13% for every drink per day, after analysis of data from 21,052 participants of the Nurses’ Health Study, Nurses’ Health Study II, and The Health Professionals Follow-Up Study [57].

As far as head and neck cancer (HNC) risk and wine consumption is concerned, no significant associations were observed in the Netherlands Cohort Study [58]. In fact, wine consumption was largely, but not statistically significantly, inversely associated with overall risk of HNC, and HNC-subtypes [58]. Similarly, wine drinking was inversely, but not statistically significantly associated with lower risk of oesophageal adenocarcinoma [59].

In stage III colon cancer patients the role of alcohol consumption in prognosis and survival was investigated by Phipps et al. [60] with surprisingly positive outcomes. Alcohol consumption was not associated with colon cancer outcomes, yet mild to moderate red wine consumption at 1–30 glasses per month of red wine was associated with longer overall survival, disease-free survival, and time to recurrence [60,61]. Similar was the result of a study by Walter et al. [62] in 3121 colorectal cancer patients that were followed up for 4.8 years. Lifetime and one-year before diagnosis abstinence from wine were associated with poorer overall survival and cancer-specific survival [62]. In addition to this, Klarich et al. [63] showed that moderate alcohol consumption was associated with a non-significant risk for colorectal cancer, while in the context of the Mediterranean diet, in which wine is the most preferred alcoholic drink, moderate alcohol intake was associated with reduced risk for colorectal cancer [63].

In cancer patients, malnutrition and weight loss is a common finding that affects response to treatment, susceptibility to treatment-related adverse events, prognosis, and quality of life [64]. Common advice is to drink a glass of wine before meals to increase appetite. Jatoi et al. [65] tested this advice in advanced cancer patients with appetite loss in a randomised controlled trial. Here, 141 patients were randomised to receive either 2 glasses of white wine or an oral nutritional supplement for 3–4 weeks. There were no statistically significant differences concerning appetite between the two groups. Hence, white wine does not improve appetite or weight in this patient group [65].

### 3.6. Association between Wine Consumption and Mental Health

The role of wine on mental health has been investigated in four studies (Table 6).

The Swedish Twin Registry study, with 12,326 participants, showed that each additional gram of alcohol over 1.16 g per day from wine was associated with a 2% decreased risk of dementia, yet the highest amount of alcohol intake from wine was associated with an increased dementia risk by 1% [66]. A cross-sectional study, used high-resolution structural MRI on 589 multi-ethnic community-dwelling elderly to assess the effect of alcohol intake and on imaging markers of brain structure. Those with light-to-moderate wine intake had larger total brain volume compared to non-drinkers, while there was a dose–response association between wine and total brain volume, indicating a protective effect of wine on brain [67].

Considering the rate of cognitive decline, its relationship with alcohol intake in middle age was investigated in the Doetinchem Cohort Study [68]. Here, 2613 participants aged 43–70 years at baseline were assessed every 5 years for 10 years. Red wine consumption was negatively associated with global cognitive function decline, memory and flexibility, with the best effect observed at 1.5 glasses of red wine per day. Since red wine was the only alcoholic beverage associated with cognitive decline indicates that non-alcoholic constituents in red wine may be responsible for this beneficial effect [68]. However, in a cohort of Alzheimer’s disease patients, who were followed-up biannually for up to 19.28 years, wine did not affect the rate of cognitive decline [69].

The PREDIMED study investigated the association between wine consumption and depression in 5505 high-risk middle aged and elderly men and women, who were followed up to seven years. Moderate wine consumption of two to seven drinks per week was significantly associated with 32% lower risk of depression, yet heavy drinking can increase the risk of depression [70]. More recently Godos et al. [71] in their observational study with 1,572 adults, found a beneficial effect of polyphenols against depression. Specifically, wine consumption was negatively associated with depressive symptoms [71].

### 3.7. Association between Wine Consumption and Gut Flora

The effect of wine polyphenols on gut microbiota has been investigated in five studies in the last five years (Table 7).

A randomised-controlled trial with 41 healthy volunteers investigated the changes in the microbial-derived phenolic metabolites of faeces, after consumption of 250 mL per day red wine for 4 weeks. Ten compounds, mainly benzoic and 4-hydroxyvaleric acids increased, after red wine intake, while the total phenolic metabolites content was also increased. Hence, a different gut microbial capacity to metabolise wine polyphenols exists among people [72]. Similarly, another analysis by the same team showed that red wine and dealcoholized red wine change the content of eight phenolic acids probably derived from the catabolism of flavan-3-ols and anthocyanins, yet alcohol does not influence the formation of phenolic metabolites by the gut flora. Inter-individual differences were also observed [73].

The impact on lipopolysaccharides’ concentrations after chronic and acute red wine intake in relation to high fat intake in middle-aged men was investigated by Clemente-Postigo et al. [74]. Ten middle-aged male volunteers were randomised to receive red wine, dealcoholized red wine, or gin for 20 days, and five adult men underwent a fat overload or a fat overload along with red wine, dealcoholized red wine, or gin. No significant differences in the change in lipopolysaccharide (LPS) or Lipopolysaccharide binding protein (LBP) concentrations with chronic red wine, dealcoholized red wine, or gin consumption were observed. *Bifidobacterium* and *Prevotella* amounts were significantly increased by red wine and were inversely correlated with LPS concentrations. There were no differences in postprandial serum LPS, LBP, or chylomicron LPS concentrations with acute red wine, dealcoholized red wine, or gin with a fatty meal. Hence, chronic red wine consumption increases *Bifidobacterium* and *Prevotella* amounts, which may have beneficial effects by leading to lower LPS concentrations [74]. Ten patients with metabolic syndrome and ten healthy subjects were included in a randomized, crossover, controlled trial. Participants consumed red wine and dealcoholized red wine for 30 days. The dominant bacterial composition did not differ significantly between the study groups after the two red wine intake periods. In the metabolic syndrome patients, red wine polyphenols significantly increased the number of faecal bifidobacteria and *Lactobacillus* and butyrate-producing bacteria at the expense of less desirable groups of bacteria such as LPS producers. The changes in gut microbiota in these patients could be responsible for the improvement in the metabolic syndrome markers. Modulation of the gut microbiota by using red wine could be an effective strategy for managing metabolic diseases associated with obesity [75].

The association between red wine intake, inflammation, and oxidative stress and faecal microbial populations was studied in 38 adult volunteers. Those who regularly consumed red wine at 100 mL per day had lower serum concentrations of malondialdehyde (MDA) and lower faecal levels of *Bifidobacterium coccoides* (*B. coccoides)*, Clostridium leptum (*C. leptum)*, *Bifidobacterium*, and *Lactobacillus*. A positive association between MDA levels and *B. coccoides* and *Lactobacillus* was also found. Thus, regular red wine intake can reduce serum lipoperoxidation, in a mechanism that involves the gut microbiota [76].

The influence of moderate red wine intake on the colonic microbiota was investigated in 15 healthy volunteers, who were classified into high, moderate, and low polyphenol metabolizers and were compared with five controls who did not drink wine. Consumption of red wine increased the global faecal microbial diversity [77].

## 4. Discussion

The most recent studies confirm the valuable role of moderate wine consumption, and especially red wine, on the prevention and treatment of chronic non-communicable diseases, such as cardiovascular disease [78,79,80], metabolic syndrome [81] and its components, cognitive decline [82,83], depression [84,85], and some cancers [86]. In the meantime, recent studies also highlight the beneficial role of red wine against oxidative stress [87] and on favour of a desirable gut bacteria [87].

More to the point, considering cardiovascular disease, studies continue to highlight the beneficial effect of wine [11,88,89]. Observational studies highlight a positive effect on cardiovascular events, mortality, and vascular inflammation in patients with established cardiovascular disease. Interventional studies show that moderate red wine intake lowers total and LDL cholesterol, as well as postprandial platelet aggregation and triglycerides in healthy participants. Considering patients with carotid atherosclerosis, patients with type 2 diabetes, patients in high cardiovascular disease (CVD) risk, and patients with hypercholesterolemia, moderate red wine consumption is associated with better blood lipid control.

In addition to this, the risk of metabolic syndrome on healthy participants and elderly on high cardiovascular disease risk is lower than on those who do not drink alcohol, while interventional studies show improvements in glycaemic and lipid control have been observed in patients with MS, type 2 diabetes, and in those in high CVD risk. In patients with type 2 diabetes also show that moderate red wine intake can reduce the number of MS constituents. The protective role of red wine on metabolic syndrome has been stated in other studies, highlighting the role of ethanol and polyphenols in modulating the endothelial nitric oxide synthase [90], while resveratrol may have protective effects against the MetS via AMP-activated protein kinase and by promoting mitochondria biogenesis [81], in addition to acting as an activator of the NAD(+)-dependent deacetylases sirtuins [81], which have been shown to extend life in animal models [91,92] and prevent insulin resistance and metabolic derangement [81].

Considering blood pressure, recent interventional studies show inconclusive results. An increase on red wine intake by women does increase blood pressure as compared to non-alcoholic wine, while two glasses of red wine increase heart rate and decrease arterial compliance after consumption, and three glasses at first decrease and then increase blood pressure (BP). In patients with diabetes, two to three glasses of red wine also increase BP (which is later lowered), yet long-term red wine intake at 1 glass with dinner per day does not influence BP in this patient population. However, in patients with mild hypertension a decrease in BP has been observed, which is attributed to the catechines and procyanidins in wine. Carolo et al. [93] highlighted that moderate wine consumption, in addition to the Mediterranean diet, may help manage hypertension [93], while Garcia-Conesa et al. [80] in their meta-analysis found that blood pressure was significantly reduced by red wine anthocyanins [80].

The association between wine consumption and risk of cancer is inconclusive, as studies show both positive and negative associations between alcohol, wine, and different types of cancer [94,95,96]. Recent studies confirm that moderate wine intake has been associated with lower risk of epithelial ovarian cancer, but higher risk of breast cancer [97,98], melanoma, and other skin cancers [57]. Additionally, recent studies show a possibly higher risk of basal cell carcinoma, yet prior evidence is inconclusive [54,99]. However, in stage III cancer patients low wine intake has been associated with longer overall survival, disease free survival, and longer time to relapse.

Considering mental health, moderate wine intake has been associated with lower risk of cognitive impairment, and greater total brain volume, whereas high amounts have been associated with greater risk of cognitive impairment. Additionally, observational studies have shown a negative association between cognitive decline and memory, attributed to the non-alcoholic part of red wine, yet no associations have been observed in Alzheimer disease patients. Orgogozo et al. [100] in their retrospective study with 3777 elderly also found an inverse association between wine intake and incident dementia, while Pasinetti [101], Pinder et al. [102] and Granzotto et al. [103] highlighted the beneficial role of wine polyphenols in the prevention of Alzheimer’s disease.

Moderate wine consumption also lowers the risk of depression in middle aged and elderly people, as well as in patients with chronic heart failure. Gea et al. apart from their study on wine and depression for the PREDIMED study [70] have also shown a positive effect of moderate alcohol intake against depression in women, in the Seguimiento University of Navarra (SUN) project [104].

As far as gut microbiome is concerned, red wine polyphenols exert prebiotic effect, while augmenting the gut flora diversity. Studies show lower lipopolysaccharide and MDA concentrations after red wine consumption, while MS patients have a more desirable microbiome after red wine consumption. Such positive effects have also been observed in prior studies [105,106].

The beneficial role of red wine has been attributed to its phytochemical compounds, as highlighted by clinical trials, where the effect of red wine has been compared to that of white wine, non-alcoholic wine, other alcoholic drinks, and water.

However, the beneficial effects of moderate alcohol consumption may not be applicable to all people, as individual characteristics and population groups may not benefit from alcohol consumption. Pregnant women belong to a group which may not benefit from alcohol intake. While alcohol concentrations in breast milk are very similar to those in maternal blood and although alcohol may cause a temporary decrease in milk yield, data on alcohol consumption during lactation are contradictory [107]. Regarding children, alcohol-related studies are scarce, as can be assessed either theoretically either by recall from adolescents or by research with children, which is difficult as the parents’ consensus reluctance on the participation of children in alcohol-related research limits this possibility. Studies have shown that early onset of alcohol consumption results in many unfavourable outcomes in both adolescence and adulthood, such as absence from classes, violent behaviour, and depression symptoms [108,109].

As the gastrointestinal tract is the first to be affected by our dietary choices, the intake of alcohol is a major factor in the encumbrance of gastrointestinal diseases. For this reason, it is encouraged to avoid alcohol consumption, although some studies report an unclear impact on the symptoms of the gastrointestinal tract from the different forms of intake [110,111,112].

Another condition to be considered is alcohol consumption in liver diseases, as studies report unclear findings regarding low to moderate consumption [113], with some studies reporting that alcohol can suppress the activity of non-alcoholic steatohepatitis by reducing the expression levels of genes involved in the immune response [114].

Another important issue is the alcohol–drug interaction, as the metabolism of a drug may increase or decrease, resulting in a change in blood levels (bioavailability) and drug half-life. Some of the classes of drugs that are affected by alcohol consumption are commonly used, for instance antihypertensives, antibiotics, antihistamines, opioid analgesics, and others [115].

Although moderate alcohol consumption as part of a healthy diet and combined with regular physical activity can be associated with beneficial health effects, high consumption and cumulative action are associated with a large number of harmful effects, affecting organs, systems and behaviour. Also, high consumption and cumulative action increases mortality, cardiovascular function, metabolic profile, and organ function, and thus initiation of alcohol consumption cannot be recommended to non-consumers [13]. As alcohol consumption has increased in recent years, the World Health Organization encourages strategies regarding the reduction of the alcohol content of alcoholic beverages in order to reduce its harmful effects [112].

It is important to highlight the limitations of the included studies. The evaluated studies included a large proportion of observational studies involving either groups of subjects or participants comparing their behaviour, diet, substance use, and exercise habits and/or based on characteristics such as sex and ethnicity. In these studies, the correlations between the potential risk factors that are beneficial or harmful from the consumption of wine and their disease outcomes over time can be identified but do not imply causation.

Another limitation concerns the gathering of information using a questionnaire and the confounding factors arising from it, such as memory recall ability, closed-ended questions, the presence or not of the researcher, and the fact that respondents often respond to what they believe the correct answer is. Furthermore, in the randomized controlled trials, heterogeneity concerning the follow-up period was observed, as well as the number of participants and the age groups.

In addition to this, most observational studies do not differentiate between white and red wine, hence, it is not possible to distinguish the part of the wine exerts beneficial role, while interventional trials study various wine amounts, and most times different amounts between men and women, and the study periods also vary, from acute consumption to weeks- and years-long consumption.

Additionally, wines differ in their composition from place to place and grape variety, and may also differ because of differences in soil, weather, climate, harvesting, wine-making, ageing, bioavailability of bioactive components, and consumed quantity. Furthermore, as supported by evidence, individual characteristics such as gut flora and genetics do play a role in the benefit exerted from wine consumption.

## 5. Conclusions

In conclusion, moderate wine intake at 1–2 glasses per day according to the guidelines [15] as part of the Mediterranean diet has been positively associated with positive effects on human health promotion and disease prevention, as well as disease prognosis. Despite this, non-drinkers should not be encouraged to initiate consumption of alcohol, while population groups that are not likely to benefit from alcohol (or wine) intake should be discouraged from drinking alcohol.

## Figures and Tables

**Table 1:** Association between wine consumption and cardiovascular disease [17,18,19,20,21,22,23,24,25,26].

Clinical Sample	Dose	Duration-Experimental Models	Main Results	References
1248 patients	Wine ≤ 500 mL/day	3.5 years of follow-up	Lower risk of cardiovascular (CV) events and mortality	Levantesi G., et al., 2013
6973 patients	1 glass of wine per day	Kansas City Cardiomyopathy Questionnaire	Better health status, lower depressive symptoms and vascular inflammation	Cosmi F., et al., 2015
449 older, U.S. male physicians with prevalent heart failure	1–2 drinks per day (beer, wine, or liquor)	7 years	Lowest mortality, independent of alcoholic drink type	Petrone A.B., et al., 2014
11,470 patients with type 2 diabetes aged at least 55 years in 20 countries	0.28 L of beer, 125 mL of wine, and 25 mL of spirits.	5 years of follow-up, self-report	Reduced risks of CV events and all-cause mortality	Blomster J.I., et al., 2014
40 otherwise healthy individuals with high cholesterol	RW, 125 mL for women and 250 mL for men/daily	1 month	Better cholesterol levels and LDL/HDL ratio	Apostolidou C., et al., 2015
23 hypercholesteraemic participants	250 mL daily of RW or WW or RO	10 weeks	Both RW and RO improved LDL oxidation lag time.	Chiu H.F., et al., 2016
12 healthy men, aged 25–39 years	4 mL/kg body weight WW or RW or ethanol solution	2 weeks	Cardioprotective effect of moderate wine consumption, independently of ethanol.	Xanthopoulou M.N., et al., 2017
157 healthy participants	RW and WW	12 months	Both RW and RO improve LDL. RW suppressed the total cholesterol	Taborsky M., et al., 2017
122 patients, aged >30 years	RW, women: 100 mL, men: 200 mL, with rich Med.	20 weeks	No changes on peak systolic, end-diastolic or mean cerebral blood flow velocity	Droste D.W., et al., 2014
122 patients, aged >30 years	RW (100 mL for women and 200 mL for men)	20 weeks	Improved the LDL/HDL ratio of the participants	Droste D.W., et al., 2013

RW, red wine; WW, white wine; RO, extract of onion; CV, cardiovascular; CHOL, cholesterol; Med., Mediterranean Diet.

**Table 2:** Association between wine consumption and blood pressure [27,28,29,30,31,32,33].

Clinical Sample	Dose	Duration-Experimental Models	Main Results	References
24 premenopausal women, aged 25–49 years	200 to 300 mL red wine (RW)/day	4 weeks	Increased the 24-h systolic and diastolic BP.	Mori T.A., et al., 2015
24 patients with well-controlled T2DM	Women: 230 mL RW/day, Men: 300 mL/day or DRW	4 weeks	RW, increased heart rate (HR), awake and asleep (24 h)	Mori T.A., et al., 2016
54 participants (age = 57 years; 85% men) with T2DM	150 mL RW at dinner/daily, with Med. diet	6 months	Reductions in BP were observed in the red wine group at midnight (3–4 h after ingestion)	Gepner Y., et al., 2016
224 patients with T2DM	150 mL of mineral water, WW, or RW with dinner	2 years	No differences were identified in blood pressure	Gepner Y., et al., 2015
18 healthy subjects, aged 25–53 years)	2 glasses of RW	24 h	Higher heart rate during the consumption and lowered after consumption	Fantin F., et al., 2016
25 normotensive men, aged 20–65 years	375 mL RW or 375 mL non-alcoholic wine or water	3 different days	A decrease in BP in the first 4 h and an increase after 20 h	Barden A.E., et al., 2013
60 untreated, hypertensive participants	2 grape extracts (grape-RW and grape alone)	4 weeks	Systolic and diastolic BP were significantly lower during the day.	Draijer R., et al., 2015

BP, blood pressure; RW, red wine; WW, white wine; DRW, dealcoholized RW; T2DM, type 2 diabetes.

**Table 3:** Association between wine consumption and metabolic syndrome (MetS) [34,35,36,37,38,39,40,41].

Clinical Sample	Dose	Duration-Experimental Models	Main Results	References
15,905 Hispanics/Latinos, aged 18–74 years	RW, WW, beer, liquor	Self-report questionnaire	Low levels of wine, related with lower odds of MetS	Vidot D.C., et al., 2016
64,046 participants aged 18–80 years	Beer, wine or spirits/mixed drinks group	Self-report questionnaire	Protective effect against MetS, and low HDL cholesterol	Slagter S.N., et al., 2014
14,375 active or retired civil servants, aged 35–74 years	Beer (350 mL), wine (120–150 mL) or spirits (40 mL).	Standard questionnaire	Consumption of wine in lesser quantities with meals was generally more protective than when taken outside of meals	Vieira B.A., et al., 2016
8103 participants (men = 2687 and women = 5416)	Red or other wines (100 mL), beer (330 mL), and spirits (50 mL)	Questionnaire consumption	Higher risk of developing specific MetS after at least 6 years of follow-up with 7 alcoholic drinks/week	Barrio-Lopez M.T., et al., 2013
5801 elderly participants at a high cardiovascular risk	100 mL of wine, 250 mL of beer, 65 mL of liquors and 32 mL of spirits	137-item FF Questionnaire	Lower prevalence of the MetS in an elderly Mediterranean population at a high cardiovascular risk	Tresserra-Rimbau A., et al., 2015
66,485 women from the French prospective E3N-EPIC cohort	150 mL wine, 250 mL beer, 70 mL fortified wine, 40 mL spirits	Questionnaires, every 2–3 years, for 14 years	Wine, associated with T2D risk, only in overweight women.	Fagherazzi G., et al., 2014
67 men at high cardiovascular risk, after a run-in period	RW (30 g alcohol/day) or gin (30 g alcohol/day)	4 weeks	Beneficial effect of the non-alcoholic fraction of RW on insulin resistance and cardiovascular disease	Chiva-Blanch G., et al., 2013
224 patients with T2DM	150 mL of mineral water, WW, or RW with dinner	2 years	Both ethanol and RW non-alcoholic constituents can be beneficial to the cardio-metabolic risk in well controlled T2D patients.	Gepner Y., et al., 2015

FFQ, Food Frequency Questionnaire; MetS, metabolic syndrome; RW, red wine; WW, white wine; DRW, dealcoholized RW; T2D, type 2 diabetes. E3N, Etude Epidémiologique auprès de femmes de la Mutuelle Générale de l’Education Nationale; EPIC, European Prospective Investigation into Cancer and Nutrition.

**Table 4:** Association between wine consumption and weight [41,42,43,44].

Clinical Sample	Dose/Type	Duration	Main Results	References
224 patients with T2DM	150 mL of mineral water, WW, or RW with dinner	2 years	No weight change in patients with T2DM	Gepner Y., et al., 2015
48 participants	150 mL of mineral water, WW or RW with dinner	2 years	Without changes to ratio of abdominal fat	Golan R., et al., 2017
14,971 from 51,529 men in the USA, aged 40–75 years	FFQ	4 years	Dose-dependent relationship	Downer M.K., et al., 2017
5879 Australian-born individuals aged 40–69 years	121-item FFQ	8 years	Greater waist circumference and body weight	MacInnis R.J., et al., 2014
7855 men aged 50–59 years	FFQ	2 years	Associated with BMI and waist circumference	Dumesnil C., et al., 2013

FFQ, Food Frequency Questionnaire; RW, red wine; WW, white wine; DRW, dealcoholized RW; T2DM, type 2 diabetes.

**Table 5:** Association between wine consumption and cancer [45,46,47,48,49,50,51,52,53,54,55,56,57,58,59].

Clinical Sample	Dose/Type/Experimental Models	Main Results	References
2513 cases of ovarian cancer	Beer, RW and WW and spirits/questionnaire	Wine consumption was associated with a lower risk of cancer	Cook L.S., et al., 2016
476,160 individuals aged 35–70 years from 10 countries	Beer, wine, sweet liquor, distilled spirits/13.9 year	No association between alcohol and UCC	Botteri E., et al., 2017
301,051 women from 10 countries	Different types of alcoholic beverages/questionnaires	No associations between alcohol consumption and endometrial cancer risk.	Fedirko, V., et al., 2013
66,481 women aged 40–65 years from the French	150 mL wine, 250 mL beer, 70 mL fortified wine, 40 mL spirits/questionnaires	>2 glasses of wine/day in postmenopausal period increased the risk of breast cancer by 33%	Fagherazzi G., et al., 2015
167,765 women from the NHS and 43,697 men	Beer, RW, WW, liquor/FFQ	Alcohol consumption is associated with increased risk of cutaneous BCC	Wu S., et al., 2015
380 BCC and 390 controls with benign skin conditions	Wine, beer, hard liquor or mixed drinks/saliva sample and questionnaires	No association between lifetime alcohol intake and early-onset BCC	Zhang Y., et al., 2014.
59,575 white postmenopausal women	Beer, wine, liquor and gin, brandy and whisky/FFQ	Increased hazard of melanoma (MM) and risk of non-melanoma skin cancer	Kubo J.T., et al., 2014
210,252 participants from the USA	Beer, RW, WW, liquor/FFQ	Alcohol intake was associated with a modest increase in the risk of melanoma	Rivera A., et al., 2016
120,852 participants aged 55–69 years from Holland	Beer, RW, WW, liquor/FFQ	Wine consumption was inversely associated with overall risk of HNC, and HNC-subtypes	Maasland D.H.E., et al., 2014
24,068 men and women aged 39–79 years	Beer, wine and spirits/FFQ	Wine drinking associated inversely with lower risk of oesophageal adenocarcinoma	Yates M., et al., 2014.
3397 patients	Beer, wine and liquor/FFQ	RW, associated with longer overall survival and disease-free survival	Phipps A.I., et al., 2016
4966 cases of incident invasive colorectal cancer (CRC)	Beer, hard cider, wine, fortified wines, hard liquor/FFQ	Wine consumption modestly associated with more favourable survival after colorectal cancer (CRC)	Phipps A.I., et al., 2017
3146 patients with CRC from southwest of Germany	Beer, wine and liquor/questionnaires	Alcohol abstinence and heavy drinking behaviour were associated with poorer survival after CRC	Walter V., et al., 2016
141 patients with incurable invasive cancer	Wine arm and nutritional supplement arm/questionnaires and diaries	Wine does not improve appetite or weight in advanced cancer patients.	Jatoi A., et al., 2016

FFQ, Food Frequency Questionnaire; RW, red wine; WW, white wine; DRW, dealcoholized RW; UCC, urothelial cell carcinoma; BCC, basal cell carcinoma; CRC, colorectal cancer; NHS, Nurses’ Health Study.

**Table 6:** Association between wine consumption and mental health [60,61,62,63,64,65].

Clinical Sample	Dose/Type/Experimental Models	Main Results	References
12,326 individuals from the Swedish Twin Registry	Beer, wine, or 6 cL of 80-proof spirits/questionnaire	>12 g of alcohol per day may increase risk of dementia.	Handing E.P., et al., 2015
589 multi-ethnic community residents of New York aged ≥65 years	Beer, wine, or liquor/FFQ	Protective effect of wine on brain	Gu Y., et al., 2014
2613 participants, aged 43–70 years	Beer, wine (red, white and rosé), fortified wine and spirits/FFQ	Only moderate red wine consumption, associated with less strong cognitive decline	Nooyens A.C., et al., 2014
360 patients with early AD in New York, Boston, Baltimore and Paris	Alcohol intake/FFQ	Wine did not affect the rate of cognitive decline	Heymann D., et al., 2016
5505 high-risk middle aged and elderly men and women	Beer, wine, spirits/FFQ	2–7 units of wine per week, associated with 32% lower risk of depression, yet heavy drinking can increase the risk of depression	Gea A., et al., 2013
1572 adults living in southern Italy	Dietary intakes of polyphenols/questionnaire	Higher dietary intake of flavonoid may be inversely associated with depressive symptoms.	Godos J., et al., 2018

FFQ, Food Frequency Questionnaire; RW, red wine; WW, white wine.

**Table 7:** Association between wine consumption and gut flora [66,67,68,69,70,71].

Clinical Sample	Dose/Type	Duration	Main Results	References
41 volunteers, aged 20−65 years	250 mL/day RW	4 weeks	The microbial metabolic profile of faeces is significantly modified after moderate intake of red wine polyphenols	Munoz-Gonzalez I., et al., 2013
60 microbial phenolic metabolites in faecal samples	DRW: 272 mL, RW: 272 mL/, Gin: 100 mL/day	3 months	The microbial metabolic profile of faeces is significantly modified after moderate intake of red wine polyphenols	Jimenez-Giron A., et al., 2013
10 male volunteers, aged 45–50 years	RW (272 mL/day), DRW (272 mL/day), or gin (100 mL/day)	20 days	Chronic RW consumption increases *Bifidobacterium* and *Prevotella* amounts, which may have beneficial effects by leading to lower plasma lipopolysaccharide (LPS) concentrations.	Clemente-Postigo M., et al., 2013
10 patients with metabolic syndrome and 10 healthy subjects	RW & DRW	30 days	Modulation of the gut microbiota by using red wine could be an effective strategy for managing metabolic diseases associated with obesity.	Moreno-Indias I., et al., 2016
38 volunteers, 55–67 years	100 mL per day RW/FFQ		Regular consumption of RW appears to be associated with a reduced serum lipoperoxidation in which the intestinal microbiota may be involved	Cuervo A., et al., 2015
41 healthy volunteers	250 mL of red wine per day	28 days	Consumption of red wine increased the global faecal microbial diversity	Barroso E., et al., 2017

FFQ, Food Frequencies Questionnaire; RW, red wine; WW, white wine; DRW, dealcoholized RW.
